# Response of Crop Yield and Productivity Contribution Rate to Long-Term Different Fertilization in Northeast of China

**DOI:** 10.3390/plants14010132

**Published:** 2025-01-04

**Authors:** Xingzhu Ma, Xiaoyu Hao, Yue Zhao, Xinhua Peng, Jinghong Ji, Shuangquan Liu, Yu Zheng, Lei Sun, Baoku Zhou

**Affiliations:** 1 Heilongjiang Academy of Agricultural Sciences, Harbin 150086, China; 2 Heilongjiang Academy of Black Soil Conservation and Utilization, Harbin 150086, China; 3 Scientific Observing and Experiment Station of Arable Land Conservation and Agriculture Environment (Heilongjiang), Ministry of Agriculture and Rural Areas, Harbin 150086, China; 4 Key Laboratory of Black Soil Protection and Utilization, Ministry of Agriculture and Rural Areas, Harbin 150086, China; 5 Institute of Agricultural Resources and Regional Planning, Chinese Academy of Agricultural Sciences, Beijing 100081, China

**Keywords:** black soil, long-term fertilization, yield sustainability, rotatioin, contribution rate of productivity

## Abstract

To reveal the changes in crop yield and contribution rate of black soil productivity under long-term different fertilization conditions in black soil areas and to find the important significance of fertilization for sustainable and stable crop yield, high yield, and improving the contribution rate of black soil nutrients. Based on the long-term experiment of black soil fertility in Harbin, the Ministry of Agriculture and Rural Affairs, under the maize–wheat–soybean rotation system, crop yield, sustainability and stability of yield, the contribution rate of black soil productivity, and natural nutrient supply capacity under 10 fertilization treatments (CK, NP, NK, PK, NPK, M, MNP, MNK, MPK, and MNPK) were analyzed. Results showed that, compared with the treatment of chemical fertilizer, yields of maize, wheat, and soybeans increased under treatment of organic fertilizer combined with chemical fertilizer, among which the yields of maize and wheat changed the most. As the rotation period lengthened, the sustainable yield index (*SYI*) values of chemical fertilizer treatment and its combination with organic fertilizer treatment gradually decreased. During the rotation period, the *SYI* value follows: chemical fertilizer combined with organic fertilizer > chemical fertilizer > organic fertilizer. The coefficient of variation (*CV*) of yield stability showed an overall trend of increasing first and then decreasing, with individual treatments showing a gradual increasing trend (NP and NPK; MNP and MNPK). Under different rotation periods, the overall contribution rate of soil productivity of long-term organic fertilizer combined with chemical fertilizer treatment was higher than that of single chemical fertilizer treatment. With the extension of the rotation period, the contribution rate of soil productivity of NPK treatment was higher and slightly increased, while other treatments showed a downward trend. Although the contribution rate of soil productivity of organic–inorganic fertilizer combined treatment (MNP and MNK) showed a downward trend, it still remained at a high level (97.2% and 95.9%). In addition, the black soil has strong phosphorus and potassium supply capacity; nitrogen was lower than those two elements, with an average natural potassium supply capacity of 94.0–97.1%. Therefore, the combination of organic and inorganic fertilizers is one of the most effective fertilization measures to stabilize crop yield in the black soil region. Nitrogen fertilizer, as a limiting factor for crop growth in the black soil region, should be emphasized in its application.

## 1. Introduction

The high, stable, efficient, and environmentally friendly production of agricultural ecosystems is crucial for national food security and sustainable development, with crop yield stability being an important component of agricultural sustainability [1]. The sustainable yield index (*SYI*) is an important parameter for measuring whether agricultural ecosystems can sustain production. The larger the *SYI*, the better the sustainability of the system [2,3]. The stability of crop yield is an important indicator for judging the quality of agricultural ecosystems, represented by the coefficient of variation (*CV*) in statistics, which can measure the degree of variation in the average yield of the same variety of crops in different years; the larger the *CV*, the lower the yield stability [4,5]. The contribution rate of soil productivity is an indicator for measuring changes in land productivity after continuous application of different fertilizers [6]; soil types and other fertilizer and tillage practices have direct, indirect, and long-term impacts on crop yield. Therefore, the evolution characteristics of crop yield and related indicators can reflect the changing trends of soil fertility and soil quality.

Long-term experiments have both temporal and climatic representativeness. Through long-term continuous observations, the impact of different fertilizers on crop yield can be accurately and objectively evaluated. Researchers have conducted many studies; firstly, nitrogen was the primary factor limiting crop yield, followed by phosphorus and potassium in tidal soil areas of China [7]. Meanwhile, crop yield and fertilization amount had a significant correlation while yield stability was not significantly related to fertilization amount but closely related to different fertilizer applications [5]. Compared with no fertilizer treatment, fertilization could increase crop yield and improved yield stability [8]. Research on the effects of fertilizer types has shown that the combination of organic and chemical fertilizers has improved the stability and potential for increased peanut yield under a rotation system of dry-land in the red soil of China [9]. However, Gao et al. (2015) [10] showed that long-term non-fertilization or biased application of chemical fertilizers weakens the stability of maize yield and reduces the contribution rate of soil productivity in the black soil region of Northeast China and the balanced application of chemical fertilizers could improve the stability and sustainability of maize yield effectively [10]. Additionally, the combination of organic and chemical fertilizer increased crop yields significantly and stabilized crop yield under crop rotation system; furthermore, there was no degradation of soil quality [10,11]. Long-term stubble and no tillage in irrigated desert soil in Gansu China improved the stability and sustainability of spring wheat yield, and intercropping could maintain the sustainable and stable production capacity of yield better than monoculture [12].

It is crucial to determine the factors of crop yield stability; the study on the effects of the different long-term fertilizers on crop yield stability in black soil areas has great significance for maintaining high and stable crop yields and sustainable agricultural development. This study is based on the long-term fertilization experiment established in 1979 to collect and analyze data on soil and crop yield under the maize–wheat–soybean rotation system, with the following aims: (1) analyzing the characteristics and stability changes in crop yield under long-term different fertilization measures in the black soil area; (2) clarifying the response of natural nutrient supply capacity of black soil; (3) providing a theoretical basis for constructing an efficient and reasonable fertilization mode and stabilizing grain yield in the black soil area of Northeast China.

## 2. Materials and Methods

### 2.1. Experimental Site

Field research was carried out at a long-term fertility experiment station (126°35′ E, 45°40′ N), Heilongjiang Academy of Agricultural Sciences, Harbin, northeast of China. The long-term experiment was initiated in 1979, which belongs to the black soil region. This region has a temperate continental monsoon climate with a mean annual temperature of 3.5 °C and a mean annual precipitation of 533 mm. The annual average sunshine hours is 2600–2800 h, frost-free period is about 135 days, and the active accumulated temperature, the sum of the daily temperature, which is over 10 °C, is about 2700 °C [13,14].

### 2.2. Experimental Design

There were ten treatments, each of them with three replicates; the plot had an area of 36 m^2^. The planting system was one crop per year, with a rotation cycle of every three years; the crops planted during the rotation cycle followed the order of wheat–soybean–maize. Maize and soybean seeds were sown in early May and harvested at the end of September, while wheat seeds were sown in early April and harvested in July. Crop samples were collected every 10 m^2^ by hand, and grain and straws were weighted separately after being air dried [15,16]. The aboveground residues of all crops (i.e., wheat–soybean–maize) were manually picked from the experimental plots after harvest. The field was plowed with a moldboard plow to a 20 cm depth; there were no herbicides or pesticides applied during the crop growing periods. The basis for selecting various crop varieties is that the crop varieties are always within the same planting area and have the same yield level.

Fertilizer amounts were shown in Table 1. Treatments included: (1) non-fertilization (CK); (2) chemical nitrogen and phosphorus combination (NP); (3) chemical nitrogen and potassium combination (NK); (4) chemical phosphorus and potassium combination (PK); (5) chemical nitrogen, phosphorus, and potassium combination (NPK); (6–10) organic manure and chemical fertilizer combination (M, MNP, MNK, MPK, and MNPK). Nitrogen (N), phosphorus (P_2_O_5_), and potassium (K_2_O) fertilizers for wheat and soybean were all applied after harvest in autumn; P_2_O_5_ and K_2_O fertilizers and half of the N fertilizer were applied as basal fertilizers for maize after harvest in autumn, and the other half of the N fertilizer was applied during the jointing stage. The manure was spread in the field before mixing it into the soil with plowing up to a 20 cm depth in the autumn. Organic fertilizer was horse manure and provided as N fertilizer at the rate of 75 kg/ha (approximately 18,600 kg manure/ha) after maize harvesting in each rotation.

### 2.3. Calculation

(1) Sustainable yield index (*SYI*) = (*Y* − σ)/*Y* max

Note: *Y* is average yield (kg/ha), σ is standard deviation of crop yield (kg/ha), and *Y* max is the highest yield of the experiment (kg/ha) [2].

(2) Coefficient of variation (*CV*) = σ/*Y*

Note: σ is standard deviation of crop yield (kg·hm^−2^) and *Y* is average yield (kg·hm^−2^) [4].

(3) Contribution of soil capacity (%) = 100% × (average yield of one or two kinds of nutrient treatment/the average yield of all nutrients treatments) [17]

(4) Nutrients spontaneous supply capacity (%) = 100% × (yield of treatment without one nutrients fertilizer/the yield of all nutrients treatments) [5,18,19]

### 2.4. Data Analyses

The yield data were analyzed by Microsoft excel 2019 and SPSS Version19.0. The *T*-test was used to test significant differences between treatments means, the significant differences between treatments were compared with the least significant difference (L.S.D.) at 5% level of probability.

## 3. Results and Analysis

### 3.1. Changes in Crop Yield Under Different Fertilization Treatments

**Figure 1:**
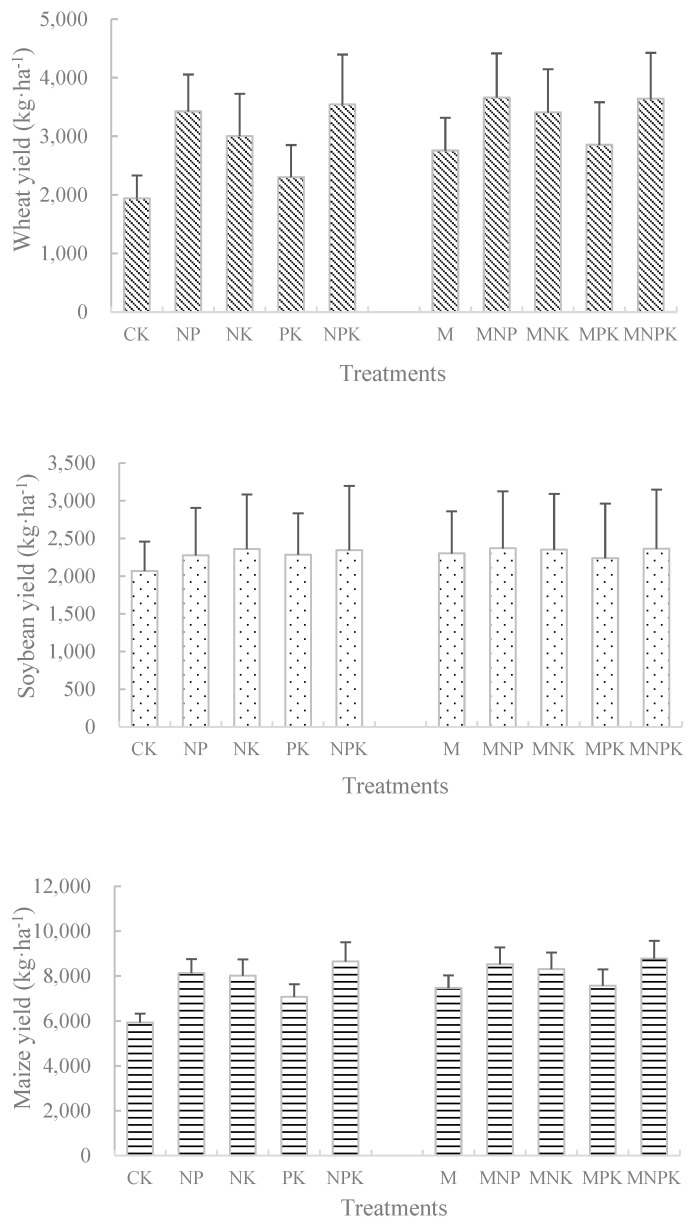
Changes in crop yield (wheat, soybean, and maize)under long-term different fertilization.

The average yield of wheat under chemical fertilizer treatments was NPK > NP > NK > PK > CK, while under organic fertilizer combination treatment, the average yield of wheat was MNPK > MNP > MNK > MPK > M (Figure 1). Compared with CK, wheat yield under fertilization treatment increased from 17.7% to 86.4%, and compared with M, wheat yield increased from 2.9% to 34.6% under treatments of chemical fertilizer combined with organic fertilizer. The change in maize yield under chemical fertilizer treatment and organic fertilizer combination treatment is consistent with that of wheat. Compared with CK, maize yield under fertilization treatment increased from 19.2% to 45.6%. Compared with M, the increase in maize yield under combined application of chemical fertilizer treatment is 1.3–17.5%. The change in soybean yield is slightly different from wheat and maize. Different fertilization treatments have relatively small changes in soybean yield. Compared with CK, the soybean yield under fertilization increased from 9.7% to 13.8%. Compared with M, the highest increase in soybean yield under the treatment of chemical fertilizer and M is 3.0%, while the yield of MPK is the lowest one.

### 3.2. Sustainability and Stability of Crop Yield

Due to the crop rotation system consisting of soybean, maize, and wheat in this experiment, in order to avoid different crops responses to fertilizers, data analysis was conducted, which was the sum of the yields of three crops for three years, to analyze the sustainability characteristics and stability of crop yields under rotation conditions (Table 2). Results showed that during the crop rotation 1st–4th (years 1–12), the *SYI* of the chemical fertilizer combined with organic fertilizer treatment was generally higher than that of the chemical fertilizer treatment. CK and PK had the lowest *SYI*; the *SYI* of CK is significantly lower than other fertilization treatments (*p* < 0.05), and the MNP and MNPK treatments had the highest *SYI*. The *CV* value of CK treatment is higher than that of other treatments, which is consistent with the sustainability results of crop yield; the *CV* values of PK, MNPK, and MPK treatments are relatively low. Overall, the differences in *CV* values among the treatments are relatively small during the crop rotation 1st–4th.

The changes in *SYI* under different fertilization treatments during the 5th to 8th rotations (years 13–24) are consistent with those in the 1st–4th rotations. After adding organic fertilizer, the *SYI* increased; the *CV* values of NK, PK and their combination with organic fertilizer, as well as the treatment of M, were higher than NP, MNP, and MNPK treatments; and the yield of NP and NPK, as well as MNP and MNPK, showed higher sustainability and stability in fertilizer treatment and organic–inorganic combination treatment, respectively.

Following the application of organic fertilizer during the crop rotation 9th to 12th (years 25–36), with the exception of the *SYI* reduction in the MNPK treatment, the *SYI* for all other treatments increased, with the CK treatment being the lowest and the NPK treatment being the highest; the PK and MPK treatments had greater yield stability (with lower *CV*), but the yields and sustainability were lower; the yield variations for the NP and NPK and MNP and MNPK treatments were consistent with those observed in the 5th to 8th rotation cycles.

With the extension of crop rotation (36 years, or 12 rotation cycles), the crop yields of CK showed a downward trend, while the yield of crops with fertilizer increased. The *SYI* values of chemical fertilizer treatment and chemical fertilizer combined with organic fertilizer treatment gradually decreased, and the sustainability of the system deteriorates. The *SYI* value of long-term NPK treatment was higher than that of single organic fertilizer treatment (M) during the crop rotation cycle, while the chemical fertilizer combined with organic fertilizer treatments was overall higher than that of chemical fertilizer treatments; the variation coefficient (*CV*) that characterizes crop yield stability is different from *SYI*, the trends were first increasing and then decreasing, and individual treatments showed a gradually increasing trend (NP and NPK, MNP and MNPK).

### 3.3. Productivity Contribution Rate of Black Soil

The productivity contribution rate is an indicator of soil productivity under different fertilizer applications [20]; the average yield of four rotation cycles (1 rotation cycle = 4 rotations) is used to study the productivity contribution of each treatment. Results showed that (Table 3) the soil productivity of MNPK and NPK remained at the same level., the overall contribution rate of soil productivity under long-term organic fertilizer combined with chemical fertilizer treatments was higher than that of single fertilizer treatments under different rotation cycles; the average values after 36 years showed the same trends. Treatments of organic fertilizer combined with chemical fertilizers had a higher soil productivity contribution rate than single chemical fertilizer applications.

With the extension of crop rotation cycles, except for the NPK treatment, which had a higher overall level of soil productivity contribution and a trend of slightly increasing, other treatments showed decreasing trends. CK and unbalanced fertilization (NP, NK, and PK) treatments decreased significantly (CK decreased by 31.0%, and unbalanced fertilization treatment decreased by an average of 12.2%). The soil productivity contribution rate of CK was the lowest one (average 68.0%), and PK followed (78.8%). In addition, CK and PK decreased to 50.7% and 69.8% under the rotation cycles of the 9th to 12th, respectively; the productivity contribution rate of PK was the lowest in the fertilization treatment. The overall changes in organic fertilizer combined with chemical fertilizer application were basically consistent with the trends of chemical fertilizer treatments, with a higher contribution rate and a lower decline rate than chemical fertilizer treatments. The soil productivity contribution rate of M was the smallest one in all the organic fertilizer treatments, and the MPK was the smallest in the combined applications of chemical fertilizer. The soil productivity contribution rates of MNP and MNK showed a decreasing trend but still remained at a high level (97.2% and 95.9%).

### 3.4. Natural Nutrient Supply Capacity of Black Soil

**Figure 2:**
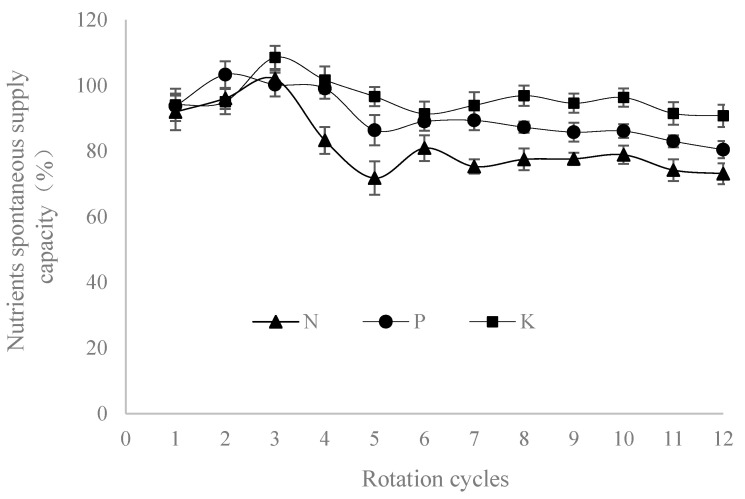
Changes in N, P, and K nutrients spontaneous supply capacity in black soil.

The natural nutrient supply capacity of soil refers to the percentage of nutrients supplied by the soil that can increase crop yield to full fertilizer yield without applying a certain nutrient when other nutrients are fully supplied [5]. Based on the yield results, changes in natural nutrient supply capacity during the rotation cycle were calculated. Results showed that (Figure 2) soil nitrogen began to decrease after the third rotation cycle, with a decrease of 9.4%, and remained stable basically between 70% and 80% after the fifth rotation cycle. The natural supply capacity of soil phosphorus averaged over 95% in the first four rotation cycles, decreased from the fifth rotation cycle, and remained above 85%. The natural supply capacity of soil potassium increased in the third cycle, then decreased and remained stable at around 94% after the fifth rotation cycle.

**Figure 3:**
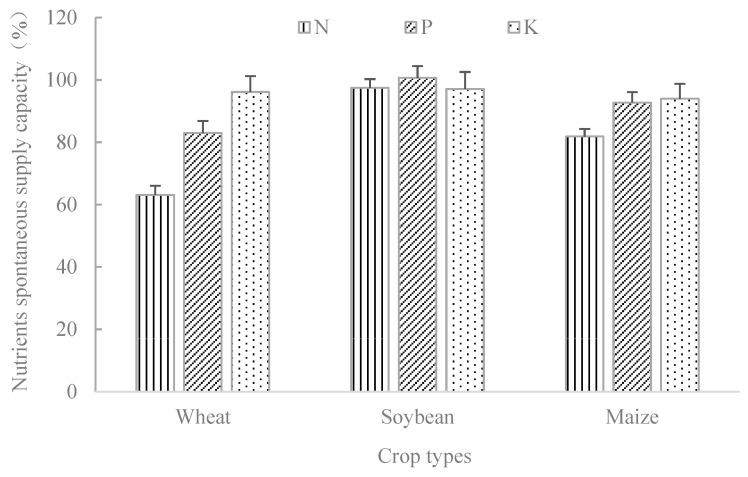
Changes in soil nutrients natural supply capacity with different crops in black soil.

The crop rotation involves wheat, soybean, and maize. In this study, the natural supply capacity of soil nitrogen, phosphorus, and potassium nutrients were analyzed according to different crops (Figure 3). Results showed that different crops have different responses to different nutrients. The natural soil nitrogen supply capacity of wheat season is about 60%, the natural phosphorus supply capacity is about 80%, and the natural potassium supply capacity is about 95%. The natural supply capacity of soil nitrogen, phosphorus, and potassium during the soybean season is above 95%. The natural supply capacity of phosphorus and potassium in the maize season soil is around 90%, while the natural supply capacity of nitrogen is lower than that of phosphorus and potassium (80%). Compared with wheat, the natural supply capacity of soil nitrogen and phosphorus is higher in soybeans and maize, while there is no significant difference in the performance of soil potassium natural supply capacity in wheat, soybeans, and maize, with an average potassium natural supply capacity of 94.0–97.1%.

## 4. Discussion

### 4.1. Effect of Long-Term Fertilization on Crop Yields

The sustainability of long-term fertilization treatment is closely related to soil type, climate, and planting system. The factors that affect yield mainly include soil organic matter, other nutrient levels, fertilizer application, crop variety, climate, soil type, and crop management [2]. Long-term applications of nitrogen fertilizer combined with phosphorus (NP and NPK) returned higher wheat yield than no fertilization (CK) and unbalanced fertilization treatments (NK and PK) in this study. Similar research results on wheat yield were obtained in Jin X. et al.’s (2018) study under the long-term fertilizer experiment of winter wheat/summer leisure system [21] and the long-term fertilization of dry-land reported by Chen Lei et al. (2006) [22]. This is due to the principle of phosphorus fertilizer promoting nitrogen metabolism, which increases the N content of crop grains in NP treatment, promotes the increase of crop yield indicators, and ultimately improves yield. Meanwhile, phosphorus fertilizer also has the ability to promote root growth, enhance crop transpiration rate, and photosynthesis. There was an increase in the diffusion rate of potassium under NK treatment, which could lead to a decrease in the absorption of calcium elements during crop growth, resulting in weak growth ability of crops and causing crop yield reduction [23].

Long-term organic fertilizer combined with chemical fertilizer could increase crop yields [2,10,24], which was consistent with the results obtained in this study; the yields of wheat, maize, and soybean under treatments of organic fertilizer combined with chemical fertilizer showed an increasing trend. This is due to the fact that although the effect of organic fertilizer was slow, it has an additive and cumulative effect after long-term application, which could promote crop growth in the next season under crop rotations. In addition, organic fertilizer contained nutrients and trace elements that were required for crop growth, enabling balanced supplementation and supply to crops and then increasing crop yields [25,26]. Therefore, the rational application of organic fertilizers and chemical fertilizers is one of the key measures to ensure grain yield and sustainable agricultural development. In addition to fertilizer factors, crop yields were also affected by other factors. The national wheat and maize yields had shown an increasing trend in the past 30 years, which was related to the widespread application of relevant agricultural technologies and measures in China, such as implementing soil testing and formula fertilization technology, promoting straw returning to the field, using high-yield varieties, and so on [27].

### 4.2. Effect of Long-Term Fertilization on Sustainability and Stability of Crop Yield

Sustainable yield index (*SYI*) is an important indicator for evaluating the sustainability of different nutrient management systems [28]. There were three crops in a rotation in this study, and each crop variety was in the same accumulated temperature zone and had consistent yield levels, which could avoid errors or impacts caused by planting different varieties. With the extension of crop rotation, the *SYI* values of all treatments showed a decreasing trend, while the *SYI* value of the CK decreased the most (about 44%). Crops in unfertilized conditions, which usually had poor stress resistance, were prone to significant fluctuations in yield [29]. The *CV* value of CK was higher than other treatments in this study, indicating that its crop yield stability is the lowest one, which is consistent with existing conclusions. Fertilization could increase *SYI* and reduce *CV* effectively through nutrient supply and supplementation, thereby reducing the impact of environmental, biological, and human factors on yield [30]. In addition, both non-fertilization and unbalanced fertilization had low yields and poor yield stability and sustainability, which was due to meteorological factors such as temperature and rainfall [28]. The lower *SYI* of unbalanced fertilization meant that its sustainability was lower than others. Chen H. et al. (2014) [29] found that the stability and sustainability of wheat yield with chemical fertilizers were significantly better than those with the application of organic fertilizers alone; that was consistent with our study, due to chemical fertilizers being able to comprehensively provide the nutrients, which is beneficial for improving crop adaptability and ensuring the stability of crop yield. The *SYI* values under long-term chemical fertilizer combined with organic fertilizer treatments in this study were higher than chemical fertilizer alone overall, which is consistent with other results. The treatments of organic–inorganic fertilizer reduced the interannual fluctuations in crop yield and was more conducive to promoting the stability and sustainability of crop yield [2,10,29].

Crop rotation also had an impact on yield; previous studies had shown that crop yield stability is higher under crop rotation than monoculture [5,31]. The yield results under the wheat–soybean–maize rotation system that was used in this study showed similar trends to existing research findings. The *SYI* with different fertilizers during the rotation cycle was higher than that of maize continuous cropping and two seasons per year [10,29]. People are generally concerned about the impact of climate on crop yields, as this may cause a reduction in photosynthesis, enhancement of respiration, and shortened nutritional growth periods of crops, thereby affecting yield trends [32]. Therefore, the impact of climate change and other factors on crop productivity should be focused on more.

### 4.3. Productivity Contribution Rate and Natural Nutrient Supply Capacity of Black Soil

Soil productivity refers to the level of soil production capacity, which is the manifestation of soil fertility under certain conditions. Changes in productivity are usually measured by the contribution rate of productivity [20]. The PK had the smallest contribution rate to soil productivity in this study, mainly due to the severe depletion of soil nitrogen nutrients; crop growth and grain formation require a large amount of nitrogen nutrients, all of which come from the soil itself. The soil productivity contribution rates of NP, NK, and NPK treatments in the results were all relatively higher (over 90%), indicating that the supply capacities of phosphorus and potassium in black soil are strong. With the extension of crop rotations, the contribution rates of soil productivity of all treatments in this study showed a decreasing trend, which is consistent with the conclusion proposed by Xu Minggang et al. (2006) [33]. Those results showed that the contribution rate of long-term fertilization to base soil fertility has been decreasing year-by-year in China The natural supply capacity of soil N was 60% to 80%, soil phosphorus remained at around 85%, and soil potassium remained at around 93%, which are different from the research results of other soil types [5]. The main reason is that the natural supply capacity of phosphorus and potassium in the black soil in Northeast China is strong, while the natural supply capacity of nitrogen is smaller than phosphorus and potassium, indicating that nitrogen is the primary factor affecting crop yield in this region.

Soybean, maize, and wheat were involved in this study; results showed that soil nitrogen had the most significant performance on soybean, followed by maize and wheat. The supply capacity of soil phosphorus was similar to nitrogen, and the natural supply capacity of soil potassium was higher, but potassium had no significant difference on wheat, soybean, and maize. Under different crop planting conditions, the natural nutrient supply capacity of black soil was different, which can be the theoretical basis for the rational fertilization.

## 5. Conclusions

Compared with long-term no fertilizer treatment, fertilization helps to increase crop yields (wheat increased by 17.7–86.4%, maize increased by 19.2–45.6%, soybean increased by 9.7–13.8%). Organic fertilizer combined with chemical fertilizer had the highest crop yield among different fertilization treatments.

With the extension of fertilization years, the sustainability index (*SYI*) value of crop yield under different fertilization treatments gradually decreases, and the system sustainability deteriorates. The *SYI* values of the combination of organic and chemical fertilizer in different rotations were higher than chemical fertilizer treatments. The variation coefficient (*CV*) was different from *SYI*, with an overall trend of first increasing and then decreasing. NP, NPK, MNP, and MNPK treatments all showed higher sustainability and stability.

The overall contribution rates of soil productivity under long-term non-fertilization (CK) and unbalanced fertilization (NP, NK, and PK) treatments decreased significantly, while the treatments of organic–chemical fertilizer combination (MNP and MNK) showed decreasing trends but still remained at a high level (97.2% and 95.9%). The soil productivity contribution of treatments of CK and PK were smaller than that of other treatments, and the supply capacities of phosphorus and potassium were strong, with an average natural supply capacity of 94.0–97.1% for potassium. We suggest that nitrogen may be one of the limiting factors for crop yield in Northeast China.

## Figures and Tables

**Table 1:** Amount of fertilizer applied in different treatments (kg/ha).

Treatment	Wheat				Soybean			Maize			1 Rotation			
	N	P_2_O_5_	K_2_O	Manure	N	P_2_O_5_	K_2_O	N	P_2_O_5_	K_2_O	N	P_2_O_5_	K_2_O	Manure (N)
CK	0	0	0	0	0	0	0	0	0	0	0	0	0	0
NP	150	75	0	0	75	150	0	150	75	0	375	300	0	0
NK	15	0	75	0	75	0	75	15	0	75	375	0	75	0
PK	0	75	75	0	0	150	75	0	75	75	0	300	75	0
NPK	150	75	75	0	75	150	75	150	75	75	375	300	75	0
M	0	0	0	18,600	0	0	0	0	0	0	0	0	0	18,600
MNP	150	75	0	18,600	75	150	0	150	75	0	375	300	0	18,600
MNK	15	0	75	18,600	75	0	75	15	0	75	375	0	75	18,600
MPK	0	75	75	18,600	0	150	75	0	75	75	0	300	75	18,600
MNPK	150	75	75	18,600	75	150	75	150	75	75	375	300	75	18,600

**Table 2:** *SYI* and *CV* of crop yield under long-term different fertilization.

Treatments	Rotations 1–4			Rotations 5–8			Rotations 9–12		
	Average Yield (kg/ha)	*SYI*	*CV* (%)	Average Yield (kg/ha)	*SYI*	*CV* (%)	Average Yield (kg/ha)	*SYI*	*CV* (%)
CK	10,354 b	0.652	10.5	10,006 c	0.582	9.1	8834 c	0.363	15.5
NP	12,259 a	0.813	5.6	12,824 b	0.753	8.2	15,799 ab	0.686	10.7
NK	12,180 a	0.804	6.2	13,465 ab	0.725	15.8	14,374 ab	0.573	18.1
PK	11,438 ab	0.775	3.7	10,672 c	0.586	14.1	12,166 b	0.561	5.3
NPK	12,300 a	0.810	6.4	14,009 a	0.814	9.2	17,131 ab	0.736	11.6
M	11,738 a	0.770	6.7	11,962 abc	0.648	15.3	13,403 ab	0.600	7.9
MNP	12,874 a	0.842	7.0	13,724 a	0.832	5.2	16,730 ab	0.706	13.3
MNK	12,218 a	0.808	6.0	13,688 a	0.769	12.2	16,329 ab	0.708	10.8
MPK	12,131 a	0.808	5.3	12,275 abc	0.628	19.9	13,224 ab	0.590	8.3
MNPK	12,578 a	0.849	4.0	14,115 a	0.829	8.2	17,425 a	0.710	16.2

Note: Different lowercase letters mean significant difference at the 0.05 level.

**Table 3:** Changes in the soil productivity contribution in different rotations.

Treatments	Contribution to the Soil Productivity (%)			
	Rotations 1–4	Rotations 5–8	Rotations 9–12	Average
CK	82.3	70.9	50.7	68.0
NP	97.5	90.9	90.7	93.0
NK	96.8	95.4	82.5	91.6
PK	90.9	75.6	69.8	78.8
NPK	97.8	99.2	98.3	98.4
M	93.3	84.7	76.9	85.0
MNP	98.4	97.2	96.0	97.2
MNK	97.1	97.0	93.7	95.9
MPK	96.5	87.0	75.9	86.5
MNPK	100.0	100.0	100.0	100.0

## Data Availability

The original contributions presented in the study are included in the article, further inquiries can be directed to the corresponding author.
